# Navigated robotic assistance results in improved screw accuracy and positive clinical outcomes: an evaluation of the first 54 cases

**DOI:** 10.1007/s11701-019-01007-z

**Published:** 2019-08-08

**Authors:** Carlo Alberto Benech, Rosa Perez, Franco Benech, Samantha L. Greeley, Neil Crawford, Charles Ledonio

**Affiliations:** 1 Department of Neurology and Clinical Neurophysiology, Fornaca Clinic, Corso Vittorio Emanuele II, 91, 10128 Turin, TO Italy; 2 Musculoskeletal Education and Research Center (MERC), A Division of Globus Medical, Inc., 2560 General Armistead Avenue, Audubon, PA 19403 USA

**Keywords:** Robotic navigated, Screw accuracy, Minimally invasive, Spine surgery

## Abstract

Computer-aided navigation and robotic guidance systems have become widespread in their utilization for spine surgery. A recent innovation combines these two advances, which theoretically provides accuracy in spinal screw placement. This study describes the cortical and pedicle screw accuracy for the first 54 cases where navigated robotic assistance was used in a surgical setting. This is a retrospective chart review of the initial 54 patients undergoing spine surgery with pedicle and cortical screws using robotic guidance with navigation. A computed tomography (CT)-based Gertzbein and Robbins System (GRS) was used to classify pedicle screw accuracy. Screw tip, tail, and angulation offsets were measured using image overlay analysis. Screw malposition, reposition, and return to operating room rates were collected. 1 of the first 54 cases was a revision surgery and was excluded from the study. Ten screws were placed without the robot due to surgeon discretion and were excluded for the data analysis of 292 screws. Only 0.68% (2/292) of the robot-assisted screws was repositioned based on surgeon discretion. Based on the GRS CT-based grading, 98.3% (287/292) were graded A or B, 1.0% (3/292) screws were graded C, and only 0.7% (2/292) screws was graded D. The average offset from preoperative plan to actual final placement was 1.9 mm from the tip, 2.3 mm from the tail, and 2.8° of angulation. In the first 53 cases, 292 screws placed with navigated robotic assistance resulted in a high level of accuracy (98.3%), adequate screw offsets from planned trajectory, and zero complications.

## Introduction

In spine surgery, cortical and pedicle screws are routinely placed for posterior spinal fixation to stabilize and promote fusion. Originally, screws were inserted via the freehand technique, which presents the risk of misplacement. Misplaced screws are not only biomechanically disadvantageous but also carry an increased risk for neurological deficit, vascular damage, and morbidity [1–3]. Advances in imaging and navigation such as fluoroscopic guidance have improved the accuracy of screw placement in the spine, yet significantly increased the amount of radiation exposure to the patient, surgeon, and operating room (OR) staff [4, 5]. In an effort to decrease the amount of radiation exposure while promoting accurate screw placement, navigated robot-assisted spine surgery was developed as a stable platform to enhance the ability of spine surgeons to perform accurate surgery.

The demonstration of improved accuracy, operative efficiency, and patient safety is required to establish the validity of this recent robotic technology. The purpose of this study is to describe the cortical and pedicle screw accuracy and screw offset for the first 54 cases in which navigated robotic assistance was used.

## Methods

This retrospective chart review was exempt from the Italian Ethics Committee. Demographic data and preoperative/postoperative computerized tomography (CT) scans of 54 patients who underwent lumbosacral pedicle screw placement with minimally invasive navigated robotic guidance using preoperative CT were analyzed.

### Navigated robot-assisted pedicle screw positioning system

**Fig. 1:**
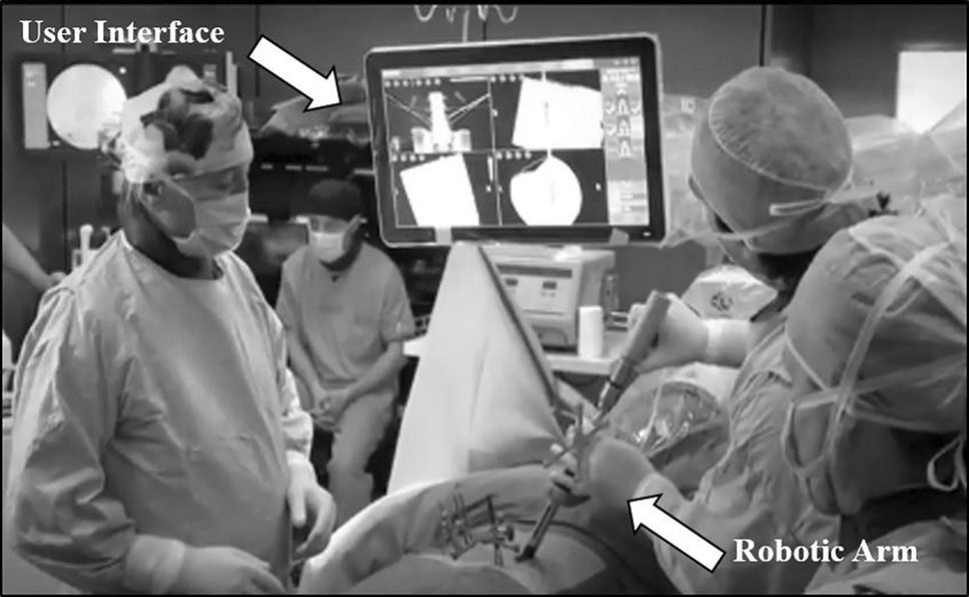
Screw insertion with the robotic positioning system

This robotic positioning system (Excelsius GPS^®^; Globus Medical, Inc. Audubon, PA, USA) (Fig. 1) uses radiological patient images (preoperative CT, intraoperative CT, or fluoroscopy), along with a dynamic reference base and positioning camera to guide pedicle screw placement in real time. In this study, the robotic system operated on one functional modality, preoperative CT.

### Preoperative CT workflow

A CT scan of the operative spinal levels was taken prior to the patient entering the OR, and screw placement planning was completed based on that scan. The CT data set was transferred into the robotic positioning system in preparation for surgery.

### Surgical technique

A dynamic reference marker was inserted into the posterior superior iliac spine, and a surveillance marker was inserted into the iliac crest for registration. The fluoroscopy registration fixture was attached to the C-arm and subsequently activated through a registration marker to ensure the robotic camera could verify its position. Fluoroscopy anteroposterior and lateral images were taken at each operative level to align the preoperative CT with the patient’s actual anatomy. Landmark checks were completed to ensure registration was calculated successfully. A surgeon-controlled foot pedal positioned the robot arm to the most cephalad planned pedicle trajectory. Stab incisions were made on the skin using a scalpel. Pedicle and cortical screws were inserted using navigated instruments guided by the robotic arm. This sequence was repeated until all screws were placed. Based on the surgeons’ discretion, 17 patients underwent a laminectomy and 8 patients underwent a discectomy. Rods were then placed and locking caps were tightened in standard fashion. Intraoperative fluoroscopy images were taken to verify screw and rod position. Screw placement was qualitatively assessed using a postoperative CT scan.

### Accuracy and screw offset

**Fig. 2:**
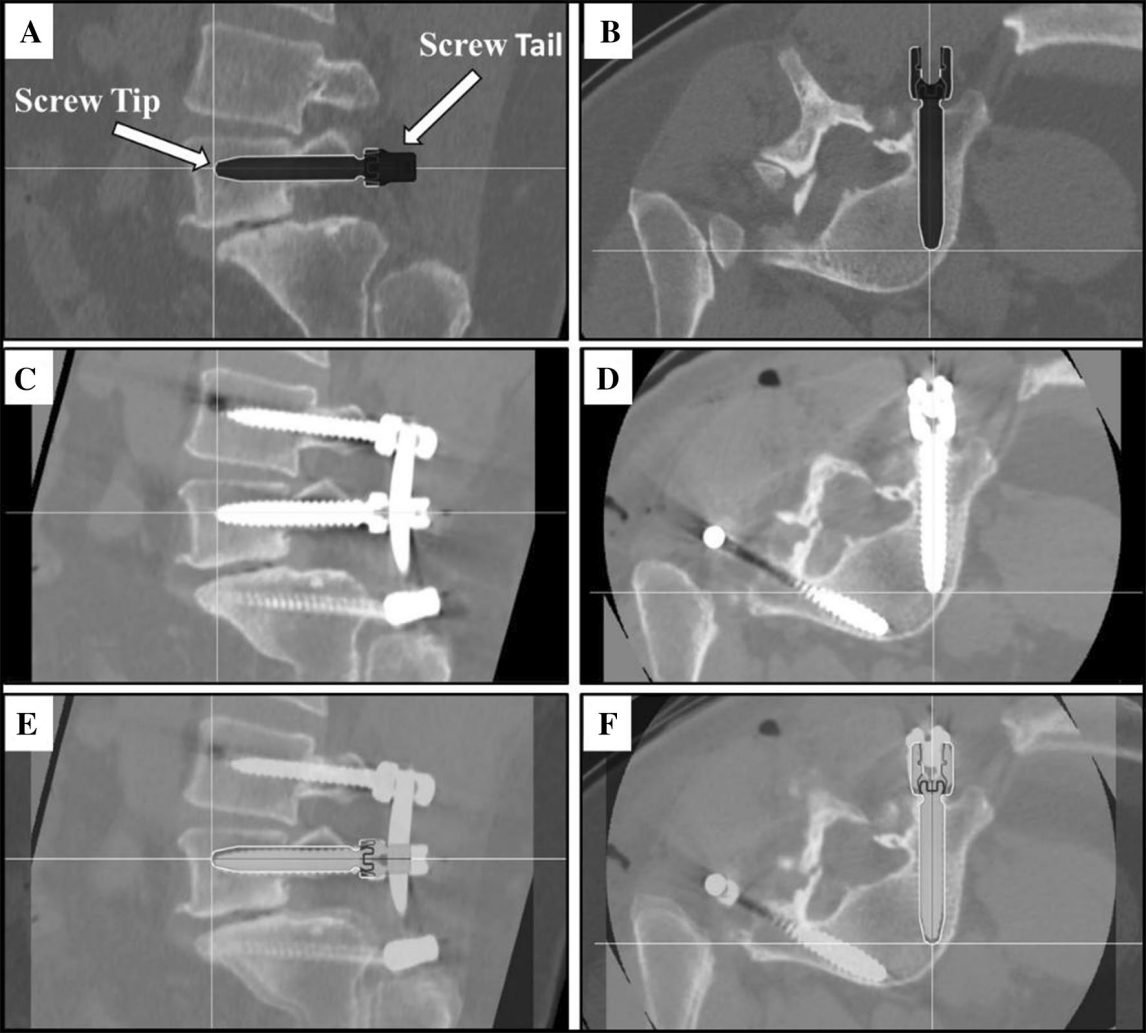
Screw tip, tail, and angle offset assessment. Right L5 screw planning in (**a**) sagittal and (**b**) axial planes. Postoperative CT of L5 screw placement without a medial or lateral breach in (**c**) sagittal and (**d**) axial planes. Image overlay analysis with preoperative planned trajectory and postoperative screw placement in (**e**) sagittal and (**f**) axial planes. The crosshairs indicate screw tip

A CT-based Gertzbein and Robbins System (GRS) [6] was used to classify pedicle screw accuracy, in which screws were graded as A (screw is completely within the pedicle), B (pedicle cortical breach < 2 mm), C (pedicle cortical breach < 4 mm), D (pedicle cortical breach < 6 mm), or E (pedicle cortical breach > 6 mm). Screws with an A or B grade were deemed clinically acceptable, while screws with a C, D, or E grade were considered inaccurate, as previously demonstrated [6–9]. The assessor was blinded to the treatment group. The number of accurate screws divided by the number of total screws placed with robotic navigation resulted in an accuracy percentage. Additionally, quantitative three-dimensional screw tip, screw tail, and screw angulation accuracies were determined using CT scans and image overlay analysis to compare preoperative planned trajectories to actual postoperative screw placement (Fig. 2). Screw trajectories were removed during image overlay to remove potential bias. Screw malposition, reposition, and return to operating room (OR) rates were collected. Intraoperative data collected included blood loss during robot use, blood loss during surgery, radiation exposure during robot use, radiation exposure time during surgery, total operative time, and screw insertion time. Blood loss of less than 25 cc was reported as no blood loss.

### Statistical analysis

Statistical analysis was performed using SPSS Statistics Version 25 software (IBM Corp., Armonk, NY, USA). Data were presented as mean ± standard deviation. Pearson correlation coefficients were calculated for the relationship between body mass index (BMI) and screw offset [10]. Fisher’s exact test was performed to determine if there was a significant relationship between two categorical variables: screw offset greater or less than 1.5 mm and clinically acceptable versus inaccurate screws according to GRS. The level of statistical significance was set to *p* < 0.05 for all statistical analysis.

## Results

### Patient population

A total of 54 patients underwent posterolateral fusion with lumbosacral pedicle or cortical screw placement with intent to use robotic guidance and navigation. One case, a revision surgery, was excluded from the study. One case was aborted due to technical difficulties and ultimately, 52 patients underwent navigated, robot-assisted surgery. These patients were diagnosed with DDD (28.9%), spondylolisthesis (34.6%), or degenerative spondylolisthesis (36.5%). The average age was 49.8 ± 11.3 years (range 23–77 years), and 29% were female. The average body mass index was 25.5 ± 4.0 kg/m^2^ (range 18.8–40.8 kg/m^2^) (Table 1).

**Table 1:** Baseline characteristics

Parameter	Overall
Number of patients	52
Gender	
Female, *n* (%)	15 (28.8%)
Male, *n* (%)	37 (71.2%)
Age, mean ± SD (range)	49.8 ± 11.3 (23–77)
BMI, mean ± SD (range)	25.5 ± 4.0 (19–41)
Diagnosis, *n* (%)	
Degenerative spondylolisthesis	19 (36.5%)
Spondylolisthesis	18 (34.6%)
Degenerative disc disease	15 (28.9%)

### Surgical data

A total of 302 screws were placed, including 12 cortical screws and 290 pedicle screws. Ten pedicle screws were placed manually due to surgeon discretion and were excluded from the study for data analysis of 292 screws. Of the 292 screws, 32.9% (96/292) were placed at L5, and 28.1% (82/292) were placed at L4. The mean operative time was 103.3 ± 42.3 min, mean estimated blood loss was 9.72 ± 42.8 cc, and mean radiation time was 17.6 ± 17.4 s (Table 2).

**Table 2:** Surgical data

Parameter	Overall
Levels treated, *n* (%)	
L2	8 (2.7%)
L3	34 (11.6%)
L4	82 (28.1%)
L5	96 (32.9%)
S1	72 (24.7%)
Mean estimated robot blood loss (cc)	0.2 ± 1.0
Mean estimated surgery blood loss (cc)	9.9 ± 43.2
Mean radiation time—robot (s)	9.2 ± 6.6
Mean radiation time—surgery (s)	17.6 ± 17.4
Mean operative time (min)	103.7 ± 42.6
Mean screw insertion time (min)	25.7 ± 14.2

### Tip, tail, and angular offset and screw accuracy

The average offset from preoperative plan to final screw placement was 1.9 ± 1.6 mm from the tip, 2.3 ± 1.6 mm from the tail, and 2.8 ± 2.3° of angulation. Based on the GRS CT-based grading, 98.3% (287/292) were graded A or B, 1.0% (3/292) screws were graded C, 0.7% (2/292) screws were graded D, and 0% screws were graded E. The GRS grades per level treated are shown in Table 3. Trends between cortical screw (*n* = 12) and pedicle screw (*n* = 280) GRS grades were similar, resulting in 83.3% A, 16.7% B, 0% C, 0% D, and 0% E for cortical screws, and 85.3% A, 12.9% B, 1.1% C, 0.7% D, and 0% E for pedicle screws. BMI was not correlated with screw tip, tail, or angular offset (tip offset: *R* = 0.19, *p* = 0.18; tail offset: *R* = 0.12, *p* = 0.41; angular offset: *R* = 0.13, *p* = 0.37). Screw offset was divided into two groups with cutoffs of 1.5 mm for tip and tail offset and 2.0° for angular offset. Of the clinically acceptable screws (Grades A and B), 49.8% and 64.8%, respectively, displayed a tip and tail offset greater than 1.5 mm. In comparison, 100% and 100% of inaccurate screws (Grade C and D, respectively) displayed a tip and tail offset greater than 1.5 mm. For angular offset, 57.1% of clinically acceptable screws (Grades A and B) had an offset of greater than 2° compared to 80% of inaccurate screws (Table 4).

**Table 3:** GRS grade per level

Level treated	Grade A	Grade B	Grade C	Grade D	Grade E
L2	4 (1.4%)	4 (1.4%)	0 (0%)	0 (0%)	0 (0%)
L3	24 (8.2%)	10 (3.4%)	0 (0%)	0 (0%)	0 (0%)
L4	58 (19.9%)	21 (7.2%)	2 (0.7%)	1 (0.3%)	0 (0%)
L5	93 (31.8%)	2 (0.7%)	0 (0%)	1 (0.3%)	0 (0%)
S1	70 (24.0%)	1 (0.3%)	1 (0.3%)	0 (0%)	0 (0%)

**Table 4:** Number of screws with tip, tail, and angular offset cutoff by grade

GRS grade	Tip offset screws > 1.5 mm	Tail offset > 1.5 mm	Angular offset > 2.0°
A	124	155	141
B	19	31	23
C	3	3	2
D	2	2	2
E	0	0	0

### Complications

Only 0.68% (2/292) of the robot-assisted screws were repositioned intraoperatively based on surgeon discretion. There were no reported adverse events, complications and no returns to the OR for misplaced screws.

## Discussion

Pedicle screw pullout strength decreases by up to 71% when the lateral wall is perforated, making accurate screw placement of utmost importance [3]. Robotic guidance with navigation assists surgeons in placing screws more consistently and accurately for posterior fixation of the spine [11–14]. The current study showed pedicle screw placement accuracy of 98%, based on GRS grading, for the first 52 patients or 292 screws placed with robotic guidance and navigation.

Few studies have presented the offset value from planned screw trajectory to final placement. Godzik et al. [15] reported tip and tail offsets in 70 pedicle screws placed with ExcelsiusGPS^®^. They found a 2.6 ± 1.5 mm tip offset, 3.3 ± 2.0 mm tail offset, and 5.6 ± 4.3° angular offset that was higher than what was found in the current study. The pedicle screw accuracy rate was 96.6% based on GRS grading, which was consistent with the 98.3% accuracy rate found in this study.

In 2015, Van Dijk et al. [9] studied the clinical accuracy and deviation in screw positions from the planning of 494 pedicle screws placed with SpineAssist™ (Mazor Robotics, Caesarea, Israel). The researchers reported 97.9% screw accuracy. In a subset of 178 screws, they found an entry point deviation of 2.0 ± 1.2 mm, an axial angular deviation of 2.2 ± 1.7°, and a sagittal angular deviation of 2.9 ± 2.4°. The study concluded that these were acceptable deviations allowing for highly accurate execution of the preoperative screw trajectory plan.

Interestingly, results from this study found no correlation between GRS grading and screw offset from planned trajectory, meaning that a screw that deviates off the planned trajectory does not constitute inaccurate screw placement. In other words, despite a deviation from the planned trajectory, pedicle screw accuracy was at 98% based on GRS grading. This emphasizes the importance of surgical proficiency while using the robot. The robot has shown to place screws in the pedicle with high accuracy, but ultimately it is up to the surgeon to plan and execute placement effectively.

The current study was not a comparative study, but rather an exploratory study on the effectiveness of navigated robotic assistance in posterior screw placement. Multiple studies have examined the screw accuracy of robot-assisted screws versus freehand screw placement with fluoroscopy guidance and found improved screw accuracy, lower radiation, improved outcomes, and fewer revisions from screw malposition with navigated robotic assistance [16–22].

In a meta-analysis by Fan et al. [16], robot-assisted pedicle screws were significantly more accurate than the conventional freehand with fluoroscopy-guided method. After comparing the accuracies of 1255 pedicle screws in the freehand group to 1682 pedicle screws in the robot-assisted group, it was concluded that the robot-assisted technique was superior to the conventional method in terms of pedicle screw accuracy.

Feng et al. [23] compared clinical outcomes of robot-assisted versus freehand fluoroscopy-assisted pedicle screw insertion, finding significantly higher 98.5% screw accuracy in the robot-assisted group compared to 91.6% accuracy in the freehand group. Similar screw placement times were found between this study by Feng et al. [23] and the current study (27.60 ± 8.58 vs. 25.7 ± 14.2, respectively). The prior study reported radiation in units of dose and number of images taken, while the current investigation reported radiation in time, making direct comparisons difficult.

In a separate study conducted by Zhang et al. [24], a robot-assisted technique resulted in significantly higher clinically accepted screw positions compared to the fluoroscopy-guided group (98.3% vs. 93.6%, respectively). Additionally, this study reported a higher rate of perfect screw position (grade A on the GRS scale) in the robot-assisted group, and fewer revisions. However, the radiation time reported during surgery in the robot-assisted group was 93.5 ± 37.9 s, significantly higher than what was found in the present study (17.6 ± 17.4 s), due to the difference in surgical workflow. In the study by Zheng et al. [24], screws were planned after an intraoperative CT scan was taken, while in the current study, a preoperative CT scan was used for planning screws. Regardless, Zheng et al. [24] reported significantly lower radiation times in the robot-assisted group than in the fluoroscopy-guided group, suggesting that if preoperative CT radiation time was accounted for in the current study, total radiation time may still be significantly lower than with a freehand technique. Similarly, a systematic review by Pennington et al. [19] found that robot-assisted surgery based on preoperative CT imaging had the least amount of patient radiation exposure and the highest amount of fluoroscopy usage compared to conventional fluoroscopy without navigation, conventional fluoroscopy with navigation, 3D fluoroscopy, and intraoperative CT-based navigation. However, these researchers reported an average fluoroscopy time of 20.1 ± 17.2 s per screw, while the current study reported a total fluoroscopy time of only 9.2 ± 6.6 s during robot use. Nevertheless, all radiation exposures presented in the review by Pennington et al. [19] were well below current safety limits.

Other studies report an average fluoroscopic exposure time anywhere from 16.8 s to 3.3 min per pedicle screw fixation case [25–28]. The current investigation reported a radiation exposure time of 17.6 ± 17.4 s for the entire case.

### Study limitations

Although this is a single-surgeon, single-site, retrospective study without comparison to a cohort, the results are consistent with findings from the literature. The method of evaluating screw offset is limited in the subjective nature of manual image overlay; however, the assessor was blinded to treatment. Future studies should report radiation exposure in both dose (mSv) and time to make comparisons with other studies easier. Larger sample sizes are needed to evaluate the effectiveness and cost–benefit analysis of this novel multi-functional robotic navigation system.

## Conclusion

This navigated robotic guidance system for percutaneous screw placement exhibited adequate screw offsets from planned trajectory, while demonstrating safety and effectiveness through a 0% return to OR rate and a 98% accuracy rate based on GRS grading, respectively. There was no correlation between GRS grading and screw offset in the studied population.
